# Supplementary material for: State-dependent signatures of anti-N-methyl-d-aspartate receptor encephalitis
Source: Brain Commun. 2022 Feb 1;4(1):fcab298. doi: 10.1093/braincomms/fcab298 (PMC8833311; doi:10.1093/braincomms/fcab298)
Supplement: fcab298_Supplementary_Data [file fcab298_supplementary_data.docx]

**Supplementary material**

**Supplementary Table 1:** Demographic variables and clinical measures of the participants**.** Table lists median and interquartilerange (IQR) of age, mRS at scan, and disease duration. Treatment, medication, and psychiatric symptoms during disease course were evaluated using a binary (present: ‘yes’ vs. absent: ‘no’) scale. Disease duration = days in hospitalization during acute phase of the disease; N = number of subjects; NMDARE = anti-NMDA receptor encephalitis.

|  |  | **NMDARE Patients** | **Healthy Controls** |
| --- | --- | --- | --- |
| **N** |  | 57 | 61 |
| **Sex** | ♀ / ♂ | 50/7 | 54/7 |
| **Age (years)** | Median ± IQR (N) | 25.00 ± 14.50 (57) | 26.00 ± 11.00 (61) |
| **mRS at scan** | Median ± IQR (N) | 1.00 ± 1.00 (55) | .. |
| **Disease duration**  **(days in hospitalization)** | Median ± IQR (N) | 62.00 ± 59.50 (52) | ·· |
| **Years between disease onset and scan** | Median ± IQR (N) | 2.43 ± 2.95 (50) | .. |
| **First-line treatment** | yes/no | 56/1 | ·· |
| **Second-line treatment** | yes/no | 28/29 | ·· |
| **Anticonvulsant medication** | yes/no | 41/16 | ·· |
| **Antipsychotic medication** | yes/no | 41/16 | ·· |
| **Positive symptoms** | yes/no | 28/29 | ·· |
| **Negative symptoms** | yes/no | 18/39 | ·· |

**Supplementary Table 2**: Location of included independent components. Component numbers, component labels, maximum t-value, MNI-coordinates of peak voxel and number of voxels in each component counting the voxels that contain the 60% highest values.

| **Somatomotor network** | | | | |
| --- | --- | --- | --- | --- |
| 6 | Postcentral gyrus (right) | 4.02 | [58 -18 48] | 2731 |
| 15 | Superior temporal gyrus (right) | 3.63 | [68 -32 10] | 1268 |
| 23 | Supplementary motor area (bil.) | 4.11 | [-2 -18 62] | 1930 |
| 44 | Superior temporal gyrus (bil.) | 3.67 | [48 0 -2] | 2814 |
| 78 | Precentral gyrus (bil.) | 4.14 | [-41 -20 63] | 751 |
| **Visual network** | | | | |
| 11 | Calcarine fissure (bil.) | 4.09 | [-10 -94 -4] | 4690 |
| 38 | Temporo-parietal-occipital junction (right) | 4.17 | [62 -46 10] | 2033 |
| 87 | Middle occipital gyrus (bil.) | 1.42 | [-40 -92 -2] | 2620 |
| 90 | Superior occipital gyrus (bil.) | 3.86 | [-22 90 34] | 1251 |
| **Subcortical network** | | | | |
| 5 | Putamen (bil.) | 4.70 | [-22 10 -12] | 2249 |
| 92 | Caudate (bil.) | 3.21 | [-12 -6 18] | 1001 |
| **Cerebellar network** | | | | |
| 7 | Cerebellum (right) | 2.77 | [46 -50 -30] | 3120 |
| **Default mode network** | | | | |
| 13 | Angular gyrus (bil.) | 4.46 | [44 -74 40] | 2222 |
| 14 | Parahippocampal gyrus (right) | 4.04 | [-23 -25 -21] | 154 |
| 24 | Dorsolateral superior frontal gyrus (right) | 5.28 | [14 46 50] | 1008 |
| 33 | Medial prefrontal cortex (bil.) | 4.37 | [- 2 62 18] | 2394 |
| 36 | Medial prefrontal cortex (bil.) | 6.34 | [-2 68 2] | 564 |
| 40 | Superior temporal gyrus (left) | 2.82 | [-54 20 -6] | 1800 |
| 59 | Hippocampus (bil.) | 3.54 | [20 -16 -16] | 1270 |
| 61 | Superior frontal gyrus, medial orb (bil.) | 3.91 | [-2 58 -12] | 882 |
| 84 | Parietal lobe, angular gyrus (bil.) | 2.69 | [-50 -60 52] | 1597 |
| 85 | Inferior frontal gyrus, opercular part (left) | 3.05 | [-62 14 18] | 1487 |
| **Dorsal attention network** | | | | |
| 10 | Parieto-occipital sulcus (bil.) | 9.20 | [-2 -60 64] | 411 |
| 41 | Postcentral gyrus (left) | 5.84 | [48 -34 62] | 563 |
| 43 | Interparietal sulcus (right) | 6.13 | [44 -50 62] | 494 |
| 45 | Precuneus (bil.) | 2.99 | [-36 -74 40] | 2768 |
| 58 | Superior parietal gyrus (bil.) | 4.33 | [38 -52 60] | 2017 |
| 74 | Superior parietal gyrus (bil.) | 4.95 | [30 -68 56] | 875 |
| 80 | Parieto-occipital sulcus (right) | 6.55 | [4 -56 72] | 260 |
| 82 | Postcentral gyrus (right) | 3.89 | [28 -46 72] | 584 |
| 86 | Postcentral gyrus (bil.) | 3.46 | [-58 -6 40] | 3042 |
| **Frontoparietal network** | | | | |
| 12 | Inferior temporal gyrus (bil.) | 3.24 | [-64 -44 -14] | 1544 |
| 28 | Middle frontal gyrus, orbital part (right) | 3.67 | [44 48 -6] | 985 |
| 29 | Middle frontal gyrus, orbital part (left) | 3.43 | [-46 50 -4] | 1674 |
| 51 | Dorsolateral superior frontal gyrus (right) | 4.22 | [28 66 6] | 322 |
| 54 | Middle frontal gyrus (bil.) | 4.13 | [32 50 38] | 1533 |
| 71 | Inferior frontal gyrus, triangular part (bil.) | 3.66 | [-56 20 32] | 1800 |
| 89 | Superior frontal gyrus (left) | 5.83 | [-24 66 17] | 575 |
| 91 | Superior temporal gyrus (left) | 1.81 | [-54 20 -8] | 5643 |

**Supplementary Table 3:** Two-way ANOVA for overall connectivity. * indicates significant effect.

|  | **Sum of squares** | **Df** | **F** | **p** |
| --- | --- | --- | --- | --- |
| Main effect: group | 0.002 | 1 | 2.52 | 0.11 |
| Main effect: state | 0.188 | 3 | 67.62 | <0.0001 * |
| Interaction effect | 0.004 | 3 | 1.58 | 0.19 |
| Residuals | 0.268 | 290 |  |  |

**Supplementary Table 4**: Average windows-wise overall connectivity (± SD) across all subjects.

|  | **Mean (**± SD**)** |
| --- | --- |
| State 1 | 0.23 (± 0.02) |
| State 2 | 0.27 (± 0.03) |
| State 3 | 0.30 (± 0.04) |
| State 4 | 0.24 (± 0.03) |

**Supplementary Table 5**: Post-hoc Kruskal-Wallis test to examine state-wise differences in overall connectivity (Chi^2^=124.37, p < 0.0001, df =3). The table contains the Bonferroni-corrected p-values for pairwise state comparison.

| **State** | ***p*** |
| --- | --- |
| State 1 - State 2 | < 0.0001 |
| State 1 - State 3 | < 0.0001 |
| State 1 - State 4 | 0.19 |
| State 2 - State 3 | 0.11 |
| State 2 - State 4 | < 0.0001 |
| State 3 - State 4 | < 0.0001 |

**Supplementary Table 6**: Two-way ANOVA for modularity. * indicates significant effect.

|  | **Sum of squares** | **Df** | **F** | **p** |
| --- | --- | --- | --- | --- |
| Main effect: group | 0.012 | 1 | 3.16 | 0.076 |
| Main effect: state | 1.113 | 3 | 98.11 | <0.0001 * |
| Interaction effect | 0.002 | 3 | 0.14 | 0.94 |
| Residuals | 1.098 | 290 |  |  |

**Supplementary Table 7**: Average window-wise modularity (± SD) across all subjects.

|  | **Mean (**± SD**)** |
| --- | --- |
| State 1 | 0.37 (± 0.06) |
| State 2 | 0.42 (± 0.07) |
| State 3 | 0.25 (± 0.05) |
| State 4 | 0.41 (± 0.07) |

**Supplementary Table 8**: Post-hoc Kruskal-Wallis test to examine state-wise differences in modularity (Chi^2^=136.08, p < 0.0001, df =3). The table contains the Bonferroni-corrected p-values for pairwise state comparison.

| **State** | ***p*** |
| --- | --- |
| State 1 - State 2 | < 0.0001 |
| State 1 - State 3 | < 0.0001 |
| State 1 - State 4 | 0.0070 |
| State 2 - State 3 | < 0.0001 |
| State 2 - State 4 | 1 |
| State 3 - State 4 | < 0.0001 |

**Supplementary Table 9:** Group differences in occurrences of states**.** Group differences were calculated using the z-test for population proportions. * p < 0.05 (uncorrected). NMDARE = anti-NMDA receptor encephalitis.

|  | **State** | **NMDARE Patients**  (N, %) | **Healthy Controls**  (N, %) | **z** | ***p_uncorr_*** |
| --- | --- | --- | --- | --- | --- |
| **Occurrence** | 1 | N=49, 85.96% | N=55, 90.16% | 0.70 | 0.48 |
|  | 2 | N=42, 73.68% | N=31, 50.82% | 2.34 | 0.019* |
|  | 3 | N=28, 49.12% | N=28, 45.90% | -0.35 | 0.73 |
|  | 4 | N=34, 59.65% | N=31, 50.82% | -0.96 | 0.34 |

**Supplementary Table 10**: Two-way ANOVA for dwell time. * indicates significant effect.

|  | **Sum of squares** | **Df** | **F** | **p** |
| --- | --- | --- | --- | --- |
| Main effect: group | 23068.00 | 1 | 6.79 | 0.0096 * |
| Main effect: state | 68622.00 | 3 | 6.73 | 0.00021 * |
| Interaction effect | 20411.00 | 3 | 2.00 | 0.11 |
| Residuals | 985147.00 | 290 |  |  |

**Supplementary Table 11**: Two-way ANOVA for transition frequencies. * indicates significant effect.

|  | **Sum of squares** | **Df** | **F** | **p** |
| --- | --- | --- | --- | --- |
| Main effect: group | 7.25 | 1 | 4.07 | 0.044 * |
| Main effect: state | 46.87 | 5 | 5.26 | <0.0001 * |
| Interaction effect | 9.20 | 5 | 1.03 | 0.40 |
| Residuals | 1239.96 | 696 |  |  |

**Supplementary Table 12**: Two-way ANOVA for fraction time. * indicates significant effect.

|  | **Sum of squares** | **Df** | **F** | **p** |
| --- | --- | --- | --- | --- |
| Main effect: group | 0.036 | 1 | 0.35 | 0.56 |
| Main effect: state | 1.515 | 3 | 4.94 | 0.0023 * |
| Interaction effect | 0.037 | 3 | 0.12 | 0.95 |
| Residuals | 29.63 | 290 |  |  |

**Supplementary Table 13:** Differences between states in dwell time (windows), transition frequencies between states (absolute numbers), and fraction time (percentage). Differences between states were calculated using a Tukey’s test. T-values and p-values are shown. * p < 0.05 (FDR-corrected), ** p < 0.01 (FDR-corrected).

|  | **State** | ***t*** | ***p_FDR_*** |
| --- | --- | --- | --- |
| **Dwell time** | 1 - 2  1 - 3  1 - 4  2 - 3  2 - 4  3 - 4 | 3.77  3.61  1.86  -0.04  -1.69  -1.61 | 0.0011 **  0.0021 **  0.25  0.99  0.33  0.37 |
| **Transition frequency** | 1 - 2 vs 1 - 3  1 - 2 vs 1 - 4  1 - 2 vs 2 - 3  1 - 2 vs 2 - 4  1 - 2 vs 3 - 4  1 - 3 vs 1 - 4  1 - 3 vs 2 - 3  1 - 3 vs 2 - 4  1 - 3 vs 3 - 4  1 - 4 vs 2 - 3  1 - 4 vs 2 - 4  1 - 4 vs 3 - 4  2 - 3 vs 2 - 4  2 - 3 vs 3 - 4  2 - 4 vs 3 - 4 | 0.00  -1.22  0.54  1.97  3.32  -1.22  0.54  1.97  3.32  1.76  3.19  4.55  1.42  2.78  1.357 | 1.00  0.83  0.99  0.36  0.012 *  0.83  0.99  0.36  0.012 *  0.49  0.019 *  0.00012 **  0.71  0.062  0.75 |
| **Fraction time** | 1 - 2  1 - 3  1 - 4  2 - 3  2 - 4  3 - 4 | 3.226  3.017  2.170  -0.093  -0.934  -0.817 | 0.0075 **  0.015 *  0.13  0.99  0.79  0.85 |

**Supplementary Table 14:** Pearson’s correlation coefficient between the participants’ average static FC and the participants’ average of each state.

|  | **R** |
| --- | --- |
| Static – State 1 | 0.94 |
| Static – State 2 | 0.91 |
| Static – State 3 | 0.87 |
| Static – State 4 | 0.71 |


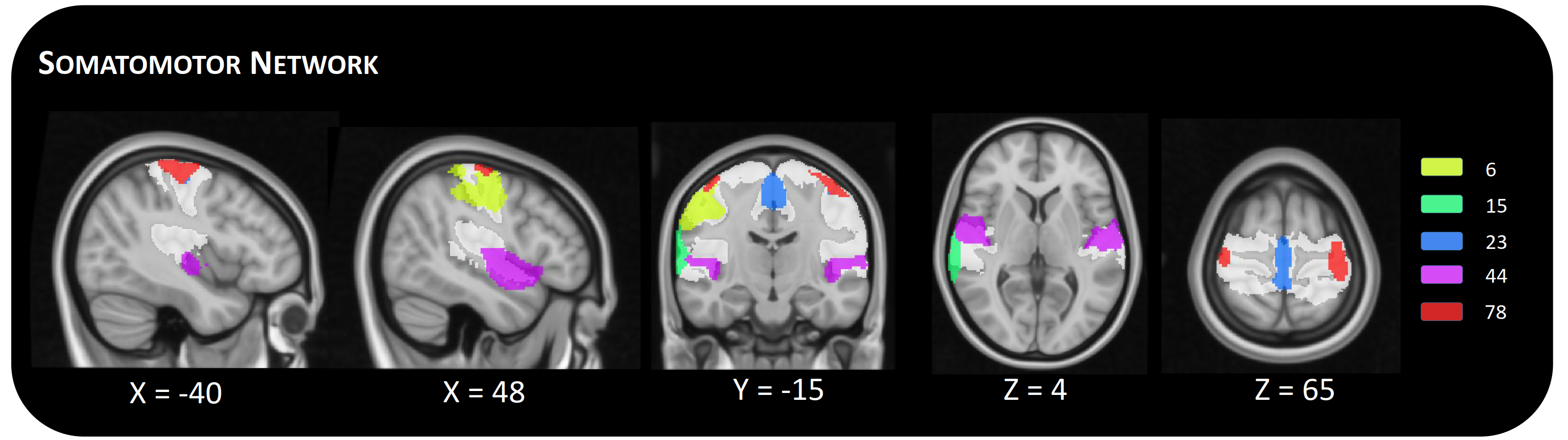


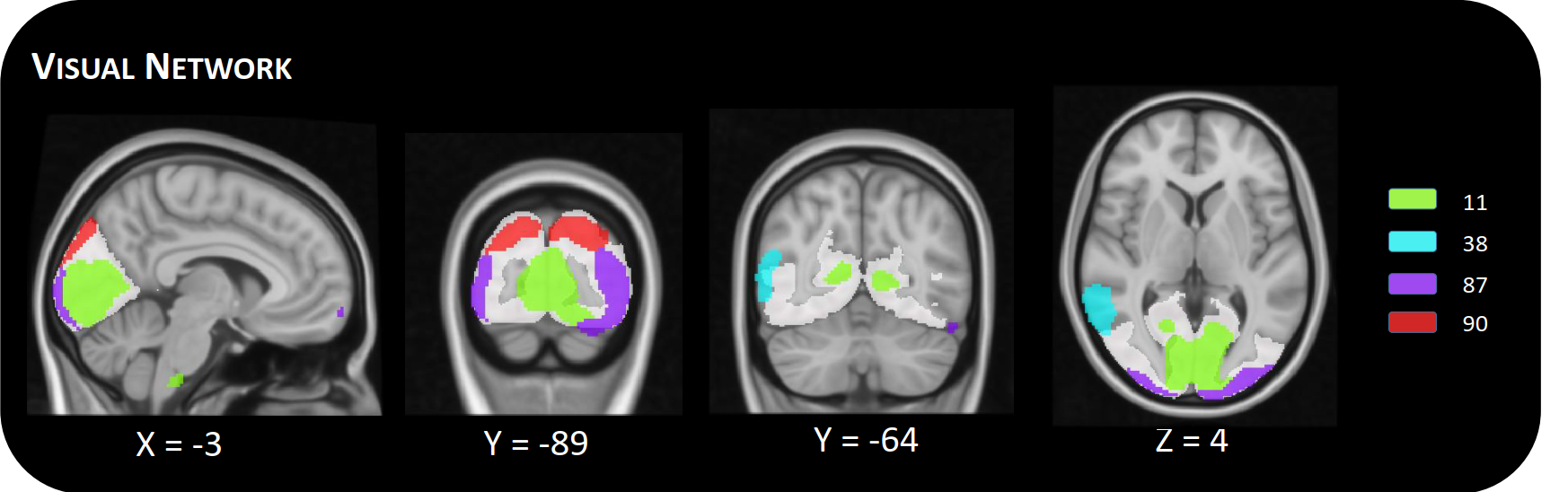


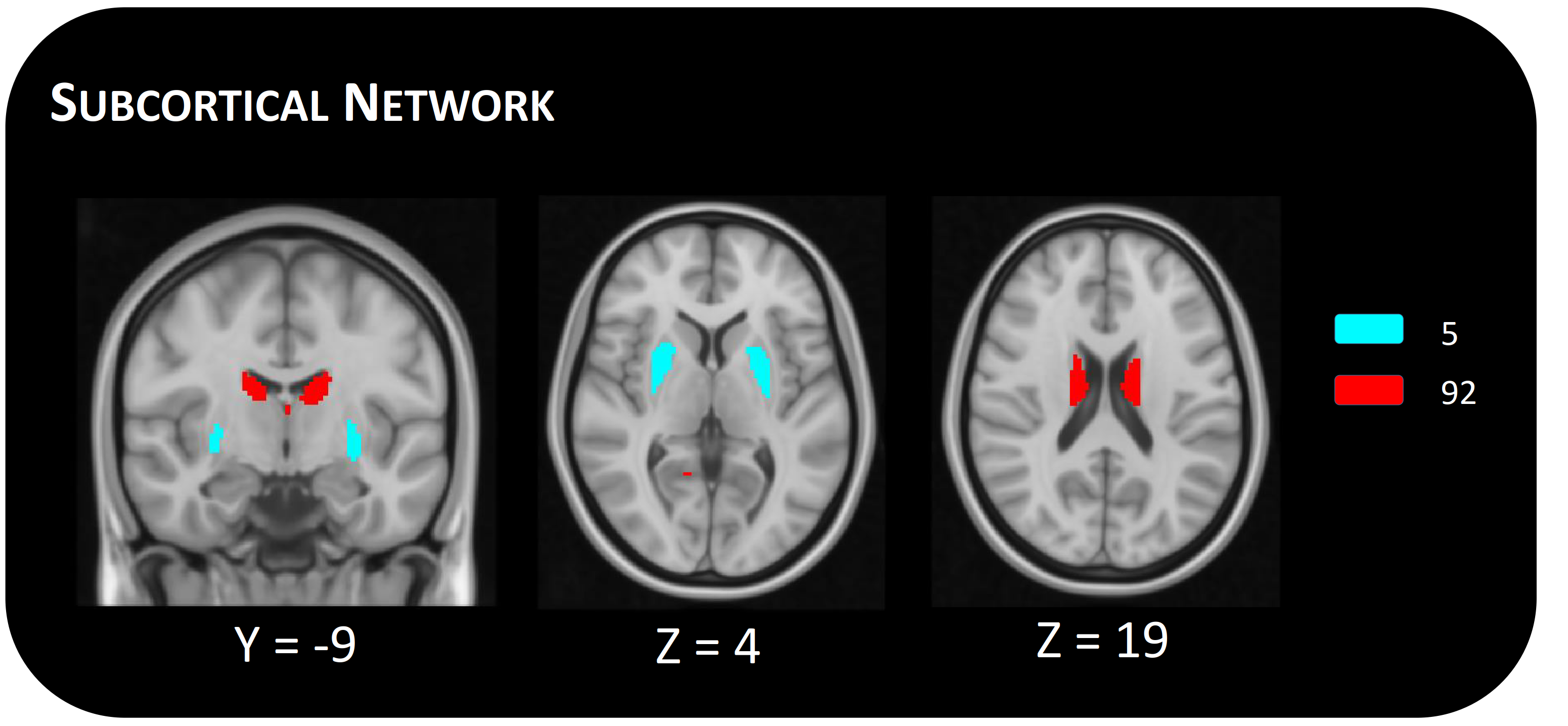


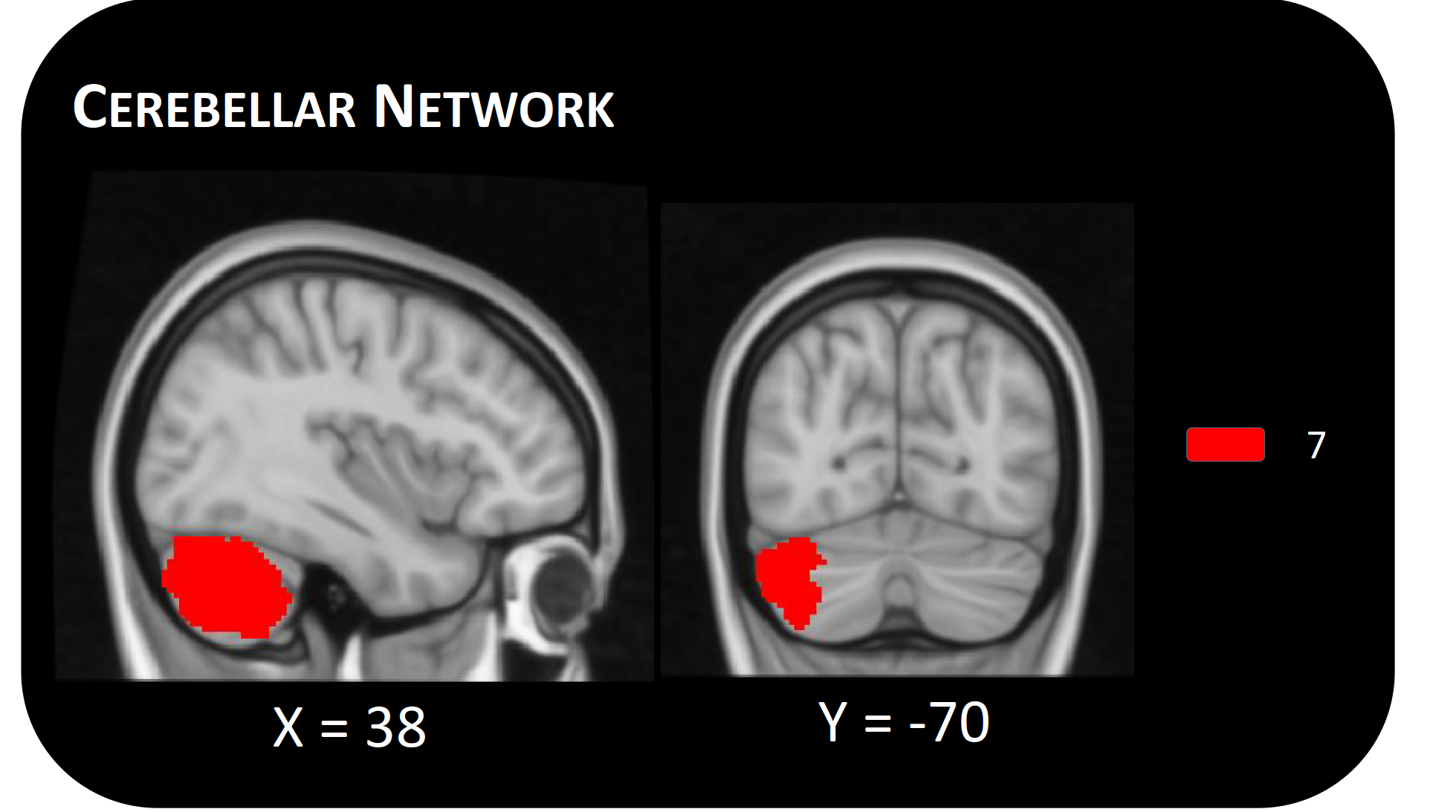


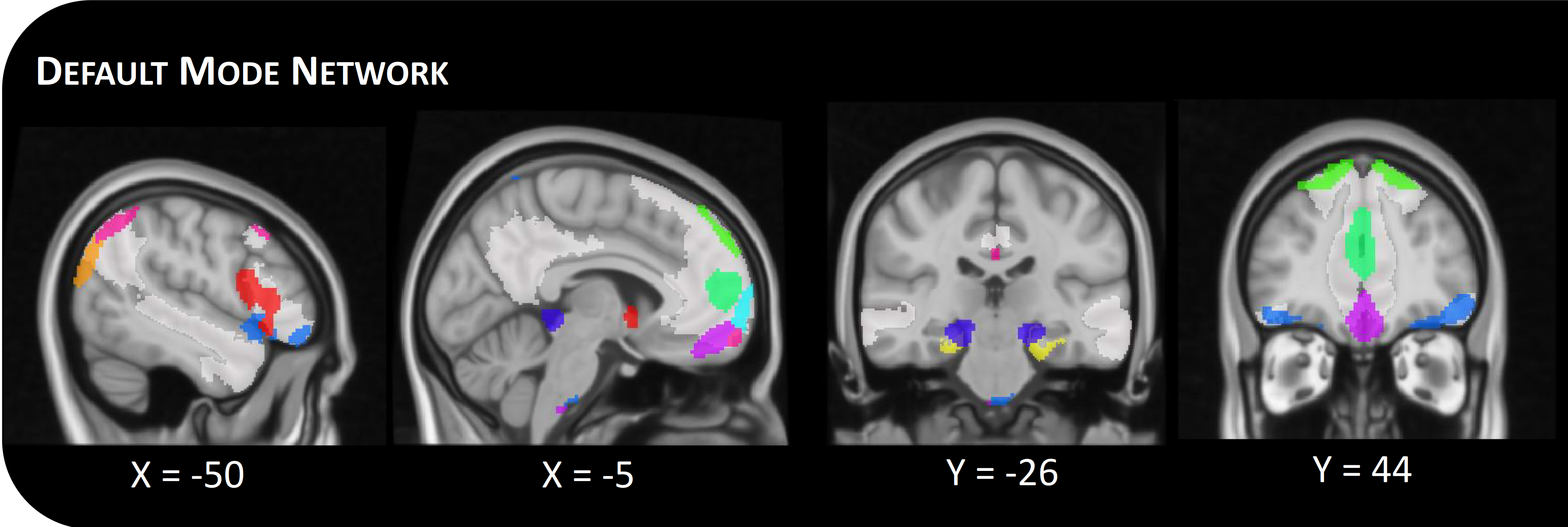

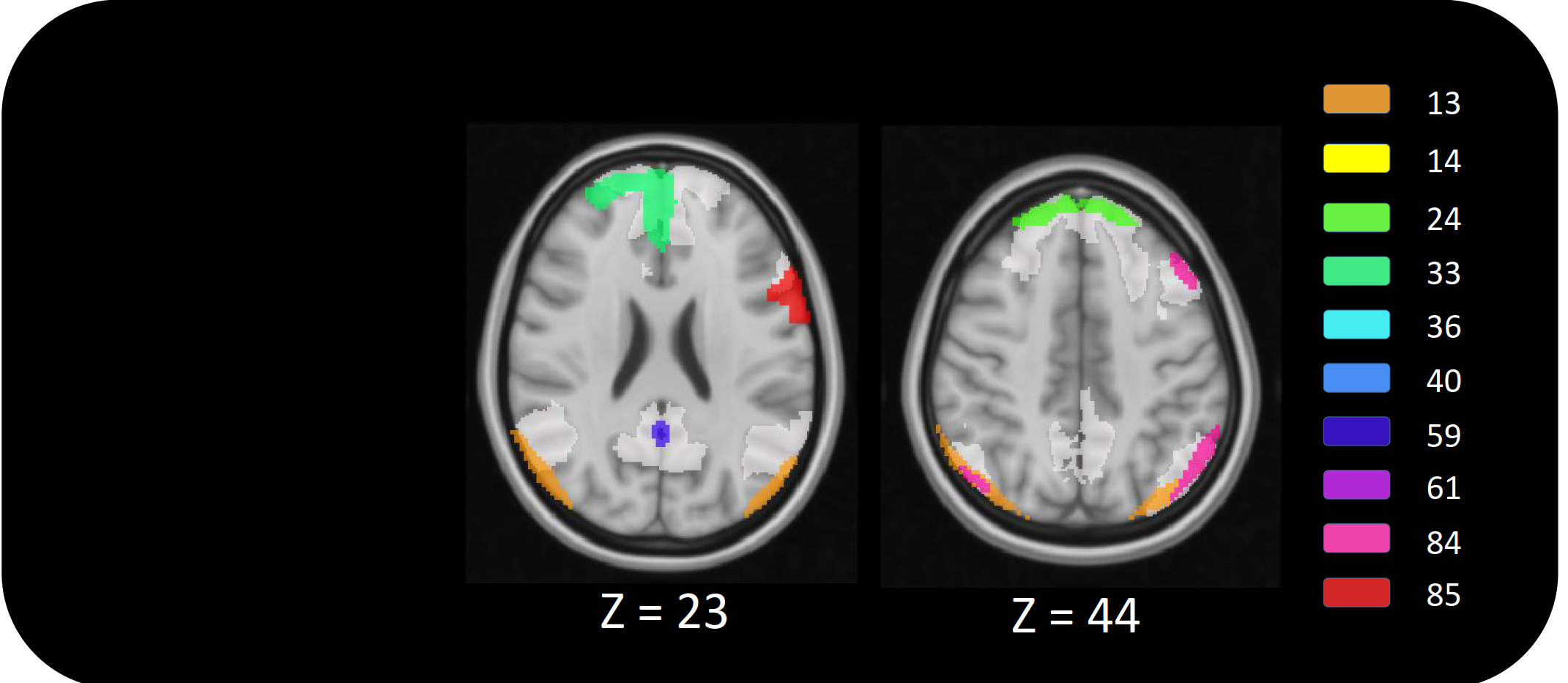


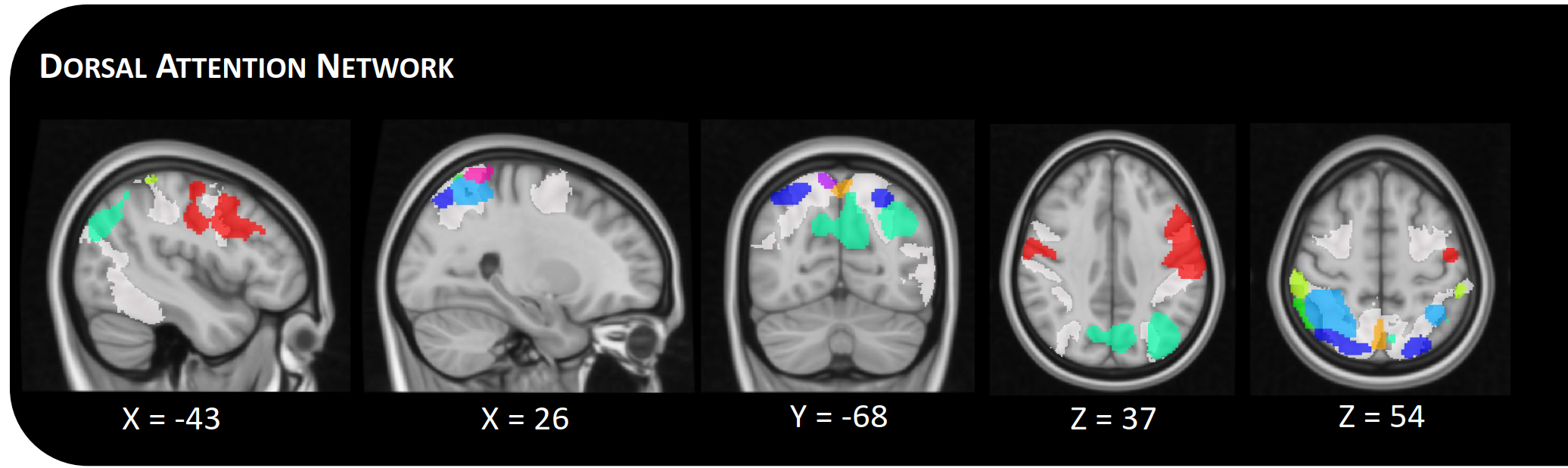

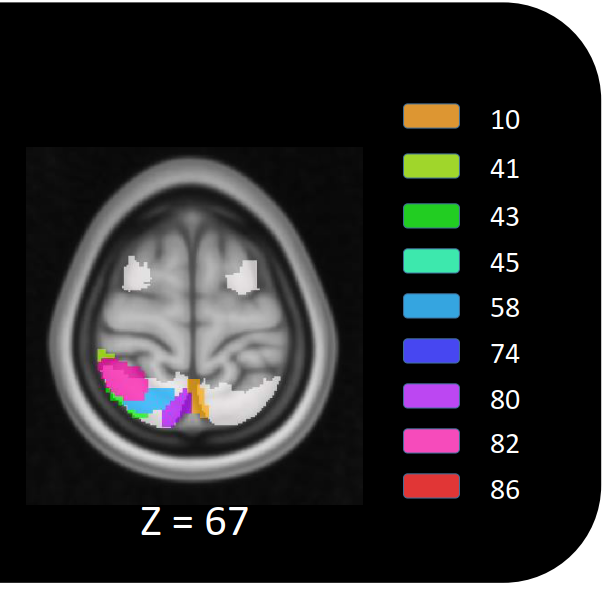


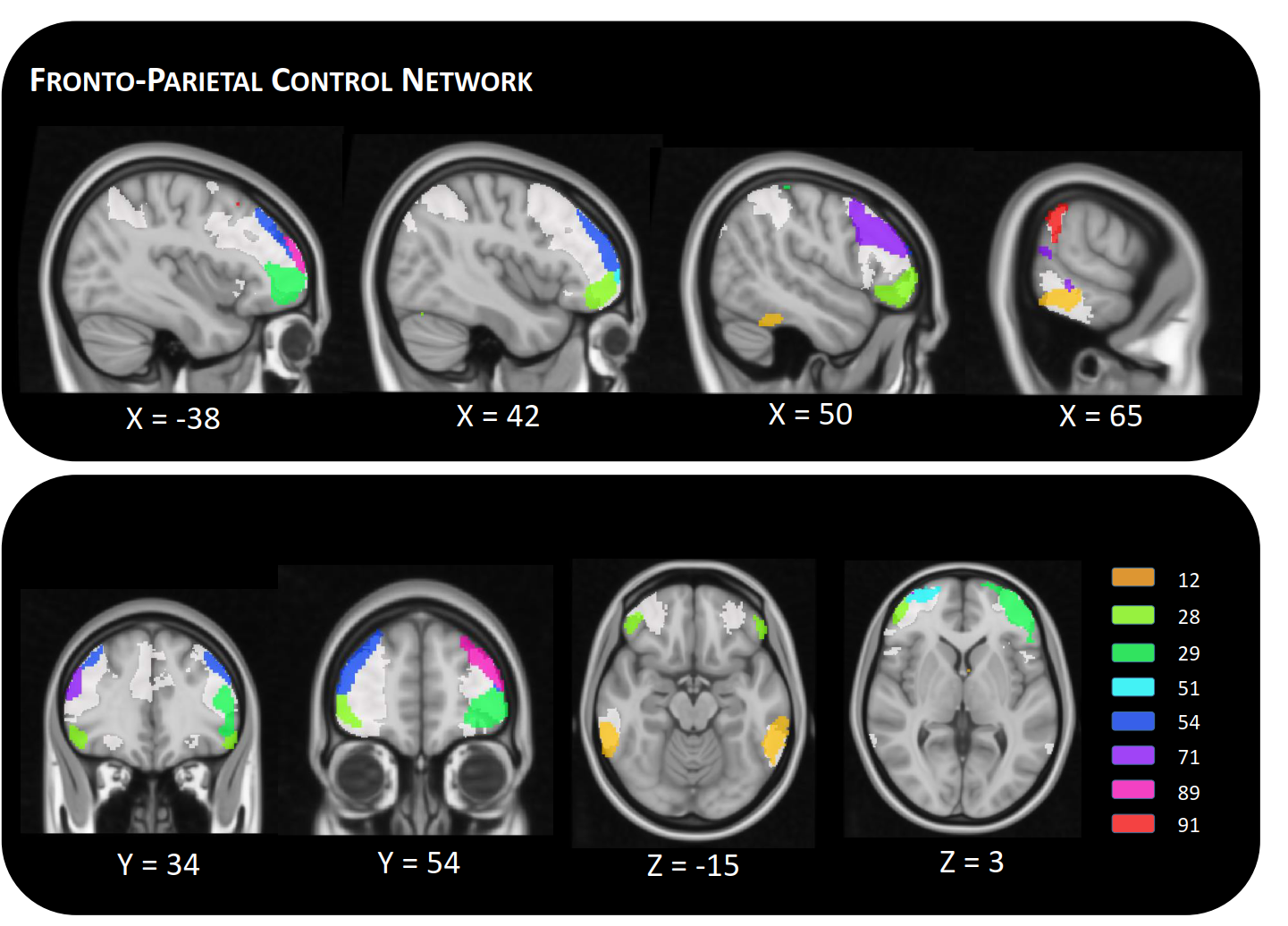


**Supplementary Fig. 1:** Included independent components in MNI space. Maps show the 39 identified signal components sorted into seven intrinsic functional connectivity networks according to ^1^, which are displayed transparent. Each color corresponds to a different component. For visualization purposes, maps show only the 60% highest values of components values. Component labels and peak coordinates are provided in Supplementary Table 2.


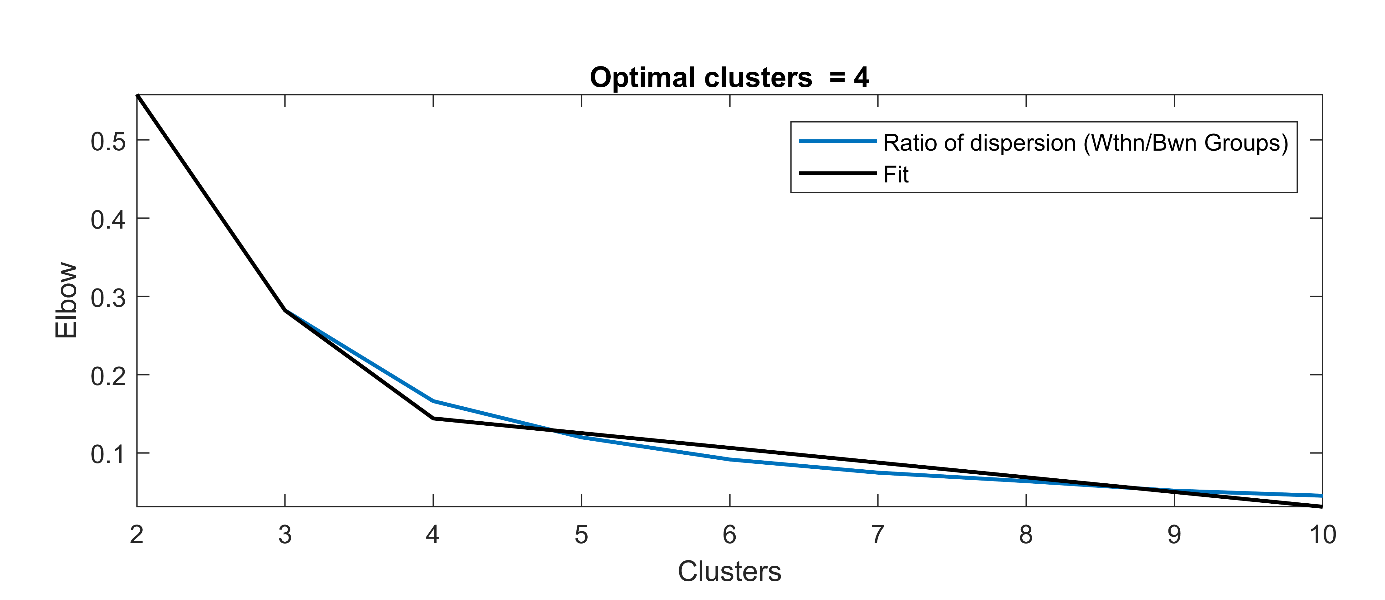


**Supplementary Fig. 2:** Visualization of elbow criterion.


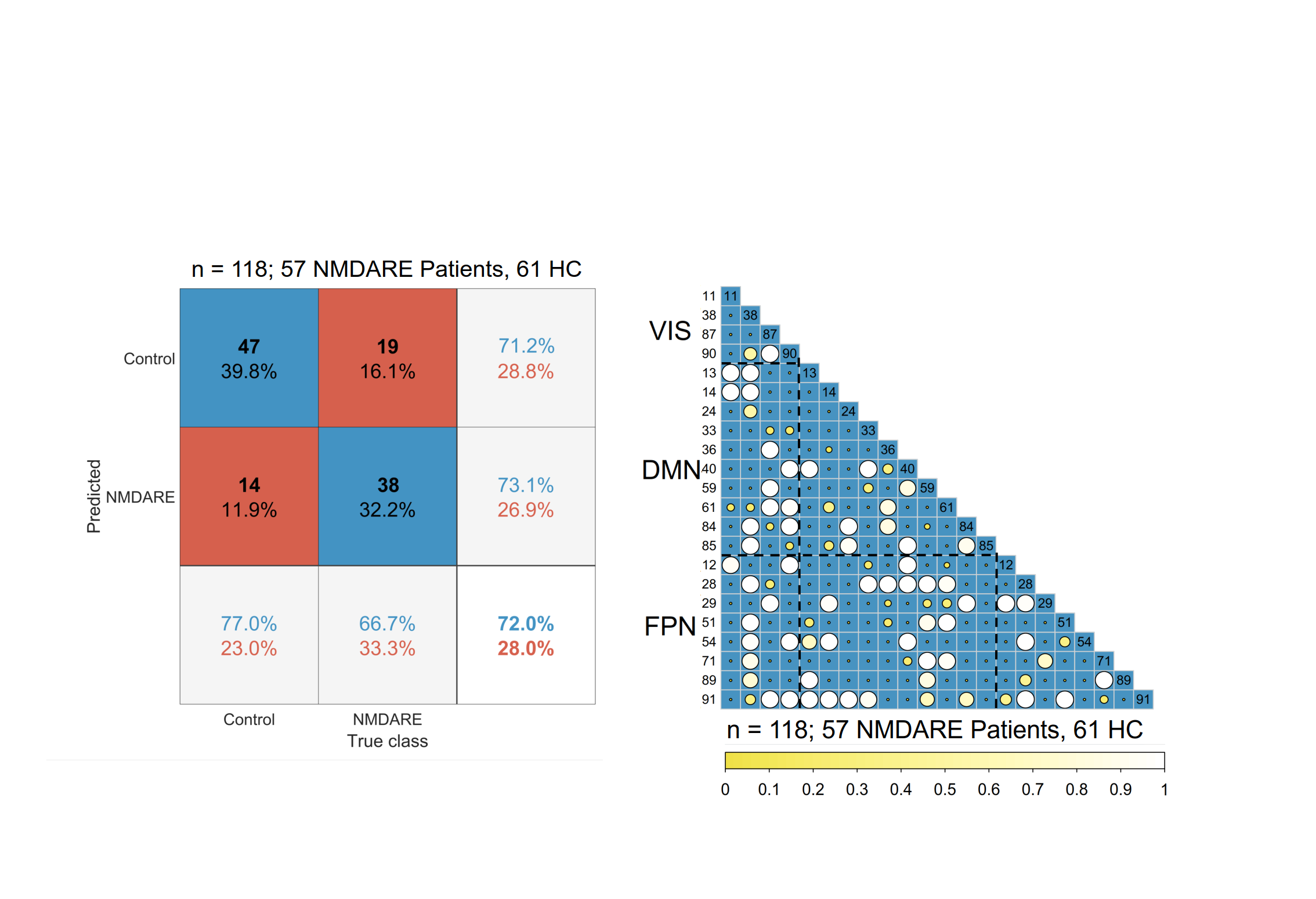


**Supplementary Fig. 3:** Confusion plot and feature selection matrix for static FC. Feature selection matrices showing all features that were selected for classification in at least 10% (threshold ≥ 0.1) of the classification after hyperparameter optimization (L1 regularization). Bigger and brighter circles indicate a higher selection rate (in percent/100) for classification. A key for the region numbers is provided in Supplementary Table 2. VIS = visual network; DMN = default mode network; FPN = fronto-parietal network; NMDARE = anti-NMDA receptor encephalitis; HC = healthy controls.


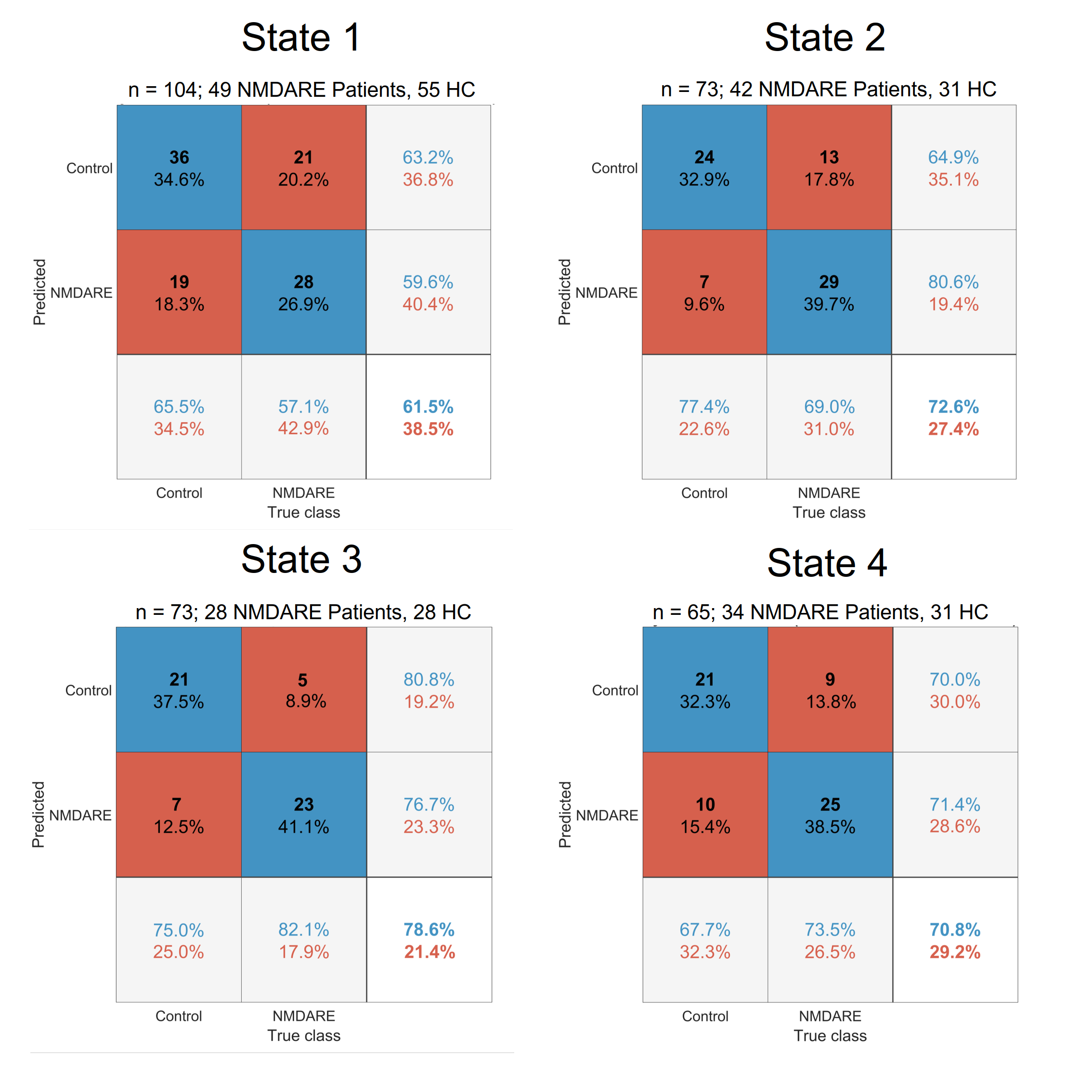


**Supplementary Fig. 4:** Standard confusion matrix for each state. Matrices indicate classification performance (i.e., true and false positive and negative rates and overall accuracy). NMDARE = NMDARE = anti-NMDA receptor encephalitis; HC = healthy controls.

**References**

1. Thomas Yeo BT, Krienen FM, Sepulcre J, et al. The organization of the human cerebral cortex estimated by intrinsic functional connectivity. *J Neurophysiol*. 2011;106(3):1125-1165. doi:10.1152/jn.00338.2011
